# Current status and future prospects of pharmacovigilance in Pakistan

**DOI:** 10.1186/s40545-019-0178-x

**Published:** 2019-05-21

**Authors:** Rabia Hussain, Mohamed Azmi Hassali

**Affiliations:** School of Pharmaceutical Sciences, Universiti Sains Malaysia, 11800 Penang, Malaysia

**Keywords:** Pakistan, Pharmacovigilance, Adverse drug reaction, DRAP

## Abstract

Countries all around the globe are working to establish robust pharmacovigilance systems. Whereas the majority of the developed countries have established well-organized pharmacovigilance systems, the developing countries still lack the basic infrastructure to establish such systems. This commentary focuses on the need of pharmacovigilance and its current status and future trends in Pakistan.

## Background

There is always a tradeoff between medicines’ side effects and the therapeutic benefits. However, evidences suggest that adverse drug reaction are very common, and may lead to hospitalization and even death [1]. To avoid adverse drug related hospitalization and mortality, an evaluation mechanism to ensure safety and monitoring of the medicines is needed [2]. The evaluation mechanism is known as pharmacovigilance, which involves detection, assessment, understanding and prevention of adverse drug effects [3]. The aim of pharmacovigilance is to identify a possible harm, which is then analyzed and investigated to minimize the hazard. This information is further communicated to the healthcare professionals and general public to improve the healthcare and patient safety [4].

### Why pharmacovigilance is important?

Medicines, during clinical trials are evaluated for their safety profile on carefully selected individuals only. However, after their release in the market, medicines are monitored during post marketing surveillance phase which relies on spontaneous reporting of adverse drug reaction (ADR) [2]. The World Health Organization (WHO) defines an ADR as any drug effect, which is noxious, unintended and undesired effect and occur at normal therapeutic doses [5]. It happens due to number of reasons, such as the limitation of scope of clinical trial data to a specific population sample, patients may experiencing co-morbidities or using other drugs, off-label use of drugs or variation in the genetic makeup of individuals [6].

Due to Thalidomide disaster in early 60s, thousands of babies were born with no or malformed limbs [7, 8]. Following this incident, in 1971, the WHO, established a program for international drug monitoring for early detection of ADRs with other member countries [9]. WHO also recommended every country to establish a national pharmacovigilance center to identify medicines which are more prone to exhibit ADRs. As a result, now member countries send report for such drugs to Uppsala Monitoring Centre (UMC), which further investigates and disseminates the necessary information globally. Currently, 134 member countries are working with UMC in Sweden including both developed and developing countries. [3]. However, 96% of the developed countries have national pharmacovigilance systems in collaboration with UMC, while only 27% of the Low and Middle Income Countries (LMICs) have such established pharmacovigilance systems. This lower number of pharmacovigilance systems in LMICs is due to lack of resources and infrastructure [10].

### Pharmacovigilance in Pakistan

Pakistan ranks 6th among the most populous country and has a population of about 207.8 million [11, 12]. In 2003, it was mentioned in the National Drug Policy of Pakistan to establish a drug monitoring and surveillance system [13]. However, the work on pharmacovigilance did not started until 2012, when an adverse drug reaction (ADR) due to a locally produced drug Isotab 20 mg (Isosorbide mononitrate, batch number J093) resulted in the deaths of more than 200 patients in Lahore [14].

Following the incident, the Supreme Court of Pakistan ordered the government to establish an independent drug regulatory authority (WHO, 2018). Thereafter, under Drug Regulatory Authority of Pakistan (DRAP) Act in 2012, DRAP was established. The DRAP serves as one of the six ministerial divisions of National Health Services Regulation and Coordination (NHSRC) to regulate the safety, quality and availability of medical devices and medicines in the country [13]. The DRAP devised a framework for post marketing surveillance of drugs with the collaboration of the United States Pharmacopoeia and Promoting Quality Medicines (USP-PQM). Drug Regulatory Authority of Pakistan (DRAP) also established a National Pharmacovigilance Centre in 2017 as well as other regional pharmacovigilance centres in the country in 2018 [15, 16]. As a result of these efforts, in 2018, Pakistan became the full member of UMC [16].

DRAP has formulated guidelines for pharmacovigilance activities and its provincial drug control unit is regularly publishing drug safety alerts based on the evidence provided through the post marketing surveillance [17]. In 2018, DRAP has organized a special training named as, “Training of Trainers, Pharmacovigilance Development of Pakistan” for the DRAP officers and focal persons from tertiary care hospitals. Furthermore, basic trainings and lectures have also been planned by DRAP to educate and train healthcare professionals about pharmacovigilance [18].

To strengthen spontaneous reporting of an ADR, the DRAP has launched an online reporting form named as “Med Vigilance” on DRAP’s official website, which is available for patients, pharmaceutical companies and healthcare professionals to report any adverse drug reaction and adverse events (DRAP, 2018). By doing so, the National Pharmacovigilance Centre (NPC) is not only fulfilling WHO’s aim of improving patient care and safety from medicine’s use perspective but also contributing towards the assessment of benefits, risks and cost- effective use of medicines [2].

### Future of pharmacovigilance in Pakistan

To promote safe use of medicines, good pharmacovigilance practices must be established to ensure the rational use of data for right purpose, however, challenges exist in the form of logistical, financial and legal constraints [19]. Pakistan needs multi-stakeholders' efforts and the standardized methods to assess severity, cause and preventability of possible ADRs. To date, there is no such data available to WHO centre for drug monitoring (UMC) about ADRs’ statistics from Pakistan, in this context, these efforts would also help the collection of this data [14]. There is also a dire need to improve communication between Pakistan Pharmacovigilance Centre and healthcare professionals, which can be done by employing various strategies such as letters to doctors, medicine alerts, newsletters, media statements, patient information leaflets and personal feedback to the ADR reporter [2]. Besides, the healthcare professions must be trained to take the lead and prioritize medication safety reporting in healthcare system involving key areas of reporting like how, when, where and what to include, while documenting an ADR. [14, 19]. With each passing day, new medicines are coming into the market, thus, stakeholders need to pay attention to pharmacovigilance in public health programmes and in the regulation of medicines to improve healthcare delivery [2].

## Conclusion

Medication safety has emerged as a great challenge to all the nations worldwide and many developed countries have pharmacovigilance systems in place to tackle this problem. The pharmacovigilance system of Pakistan is still at its infancy, and many reforms have been introduced by the government body to improve the system. However, the pharmacovigilance system requires a major revamp, which includes a multi-stakeholders’ approach and standardization of methods to address medicines safety issues in the country.

## Abbreviations

ADR: Adverse drug reaction
DRAP: Drug Regulatory Authority of Pakistan
UMC: Uppsala Monitoring Centre
WHO: World Health Organization
